# A Conditioned Medium of Umbilical Cord Mesenchymal Stem Cells Overexpressing Wnt7a Promotes Wound Repair and Regeneration of Hair Follicles in Mice

**DOI:** 10.1155/2017/3738071

**Published:** 2017-02-27

**Authors:** Liang Dong, Haojie Hao, Jiejie Liu, Dongdong Ti, Chuan Tong, Qian Hou, Meirong Li, Jingxi Zheng, Gang Liu, Xiaobing Fu, Weidong Han

**Affiliations:** Institute of Basic Medicine Science, College of Life Science, Chinese PLA General Hospital, Beijing 100853, China

## Abstract

Mesenchymal stem cells (MSCs) can affect the microenvironment of a wound and thereby accelerate wound healing. Wnt proteins act as key mediators of skin development and participate in the formation of skin appendages such as hair. The mechanisms of action of MSCs and Wnt proteins on skin wounds are largely unknown. Here, we prepared a Wnt7a-containing conditioned medium (Wnt-CM) from the supernatant of cultured human umbilical cord-MSCs (UC-MSCs) overexpressing Wnt7a in order to examine the effects of this CM on cutaneous healing. Our results revealed that Wnt-CM can accelerate wound closure and induce regeneration of hair follicles. Meanwhile, Wnt-CM enhanced expression of extracellular matrix (ECM) components and cell migration of fibroblasts but inhibited the migratory ability and expression of K6 and K16 in keratinocytes by enhancing expression of c-Myc. However, we found that the CM of fibroblasts treated with Wnt-CM (HF^Wnt-CM^-CM) can also promote wound repair and keratinocyte migration; but there was no increase in the number of hair follicles of regeneration. These data indicate that Wnt7a and UC-MSCs have synergistic effects: they can accelerate wound repair and induce hair regeneration via cellular communication in the wound microenvironment. Thus, this study opens up new avenues of research on the mechanisms underlying wound repair.

## 1. Introduction

The skin is the largest organ and functions as a protective barrier against aggression of external microorganisms and dehydration [1]. Skin injury is very common and is associated with a high rate of mortality and morbidity because this type of injury not only abrogates the barrier function of the skin but also alters the perception of pain and temperature and the sense of touch [2, 3]. In humans, problems with wound healing can manifest themselves as delayed wound healing (e.g., in diabetes or radiation exposure), excessive healing (e.g., hypertrophic or keloid scars), or a lack of skin appendages (e.g., hair follicles or sweat glands) [4]. Previously, wound closure as early as possible was the main goal of treatment. With the increasing demand for quality repair of damaged skin, restoration of the anatomy and functions of the skin after wounding (to achieve perfect healing) has become the main focus in the field of wound repair [5, 6]. Thus, there is a need to identify an effective approach to enhancement of wound healing.

Cutaneous wound repair is similar to embryonic skin development in many respects and represents an attempt to restore integrity of the injured tissue [7–9]. However, in the middle and late phases of wound healing, cellular interactions are dominated by the interplay of keratinocytes and fibroblasts; these events gradually shift the microenvironment away from an inflammatory one to a microenvironment that promotes the formation of granulation tissue [10]. Fibroblasts are an important component of the skin and play a crucial role in wound repair by constructing and maintaining the extracellular matrix (ECM) through the production of matrix proteins, metalloproteinases, and relevant inhibitors [11]. However, these properties change in different microenvironments. Studies have shown differences between fibroblasts during development and in adult skin and between undamaged and wounded skin, including differences in cell migration and in the expression of cytokines and growth factors. Under conditions of hair follicle development, the underlying dermis fibroblasts send signals to the epidermis to form a placode and to induce formation of hair follicles [10, 12]. On the other hand, adult skin does not normally show regeneration of hair follicles, but an exception has been documented in the case of adult mouse skin in response to transgenic Wnt/*β*-catenin or to wound-induced epidermal activation of Wnt/*β*-catenin [13]. Thus, a change in the microenvironment plays a key role in hair follicle regeneration. Regenerated hair follicles contain structures called dermal papillae (DP), implying crosstalk between the Wnt-activated epidermis and the dermis in the microenvironment of the wound. Thus, provision of an appropriate microenvironment for wound healing may enhance the crosstalk among cells and between cells and cytokines to achieve the goal of perfect repair.

Mesenchymal stem cells (MSCs) can create a favorable microenvironment for tissue regeneration through the secretion of a variety of prosurvival and promigratory cytokines and growth factors after MSC transplant [14–16]. Research shows that MSC-conditioned medium (MSC-CM) can enhance migration of fibroblasts and keratinocytes according to scratch assays in vitro [17]. Another study showed that MSC-CM by providing a type of pigment epithelium-derived factor (PEDF) can stimulate migration of fibroblasts to injured tissues; these cells release cytokines and ECM molecules modulating the growth of parenchymal cells and scar healing [18]. Furthermore, another study showed that MSC-CM can decrease high glucose (HG) and/or lipopolysaccharide- (LPS-) induced overproduction of reactive oxygen species and activation of the extracellular signal-regulated kinase (Erk) signaling pathway, thereby promoting proliferation and migration of keratinocytes by creating a diabetes-like microenvironment [19]. Thus, depending on various disease-related microenvironments, preconditioning of MSCs by physical, chemical, and genetic methods is necessary to maximize their therapeutic potential in wound repair.

Wnt proteins are key mediators of skin development and participate in various processes, from the development of the dermis to formation of skin appendages such as hair [20, 21]. The studies have shown that hair follicle neogenesis can be induced in 1 cm^2^ (or larger) full-thickness wounds in mice: further, when Wnt7a is overexpressed, the number of hair follicles in the wound increases [13]. Thus, Wnt signaling in the process of wound repair may provide a microenvironment similar to that present at the developmental stage of the skin; several lines of evidence indirectly support a role for Wnt signaling in cutaneous repair [22]. In contrast, an inhibitory effect of *β*-catenin on reepithelialization has been suggested in a study that showed enhanced epidermal nuclear accumulation of *β*-catenin at the edge of chronic ulcers and that pharmacological stabilization of *β*-catenin inhibits keratinocyte migration by increasing c-Myc expression [23]. However, the combined effects of Wnt and MSCs in wound repair are still unclear.

Here, we prepared CM from the supernatant of cultured Wnt7a-overexpressing umbilical cord- (UC-) MSCs (Wnt-CM) and evaluated its effect on cutaneous healing in mice. Our results suggest that Wnt-CM can enhance the expression of ECM components and cell migration of fibroblasts and thereby accelerate wound closure and hair follicle regeneration, but it inhibits the migratory ability of keratinocytes. In contrast, HF^Wnt-CM^-CM (fibroblasts treated with Wnt-CM) can accelerate wound closure and promote keratinocyte migration; however, the number of hair follicles did not increase compared to the control group. These results indicate that Wnt7a overexpression by UC-MSCs can enhance wound repair and induce hair regeneration through changes in the wound microenvironment that affect cell-cell interactions.

## 2. Materials and Methods

### 2.1. Ethics Statement

Human UC samples were obtained from a hospital after delivery of full-term infants. Human skin samples were selected by experienced plastic surgeons and collected during reconstructive procedures. All human skin tissues and human UCs were collected according to the protocol approved by the Ethics Committee of the Chinese PLA General Hospital, and informed consent forms were signed by the donors.

Six-week-old male C57BL/6 mice were obtained from the Chinese PLA General Hospital. The protocol of animal experiments was approved by the medical Ethics Committee of the Chinese PLA General Hospital.

### 2.2. Amplification and Purification of Retroviral Vectors

Human Wnt7a cDNA (NM_004625, Origene, BJ, CHN) was inserted between the* Mlu*I and* Sal*I sites of a retroviral vector (PWPT, from Shanghai Institute of Biochemistry and Cell Biology; researcher Xin Wang). Recombinant vectors were amplified in HEK 293T cells and then purified using polyethylene glycol (PEG) 6000 by the precipitation method. The viral particles expressing Wnt7a were resuspended in phosphate-buffered saline (PBS) and stored at −80°C.

### 2.3. Isolation of UC-MSCs and Preparation of Wnt-CM

Wharton's jelly from UCs was excised, minced into pieces of 0.5–1.0 mm^3^ using scissors and scalpels, and washed twice with PBS. UC-MSCs were isolated and harvested as previously described [24]. The cells were then purified and identified. Passage-3 MSCs were cultured to 50–60% confluence in T-75 culture flasks and incubated with a medium containing the Wnt7a-expressing virus combined with polybrene (8 mg/mL). This medium was added to the flask, replenished with 12 mL of serum-free DMEM (Gibco, NY, USA) and incubated for 24 hours prior to harvesting of the CM of the cells infected with the Wnt7a-expressing virus (Wnt-CM). The culture supernatant was collected from passage-3 UC-MSCs and named MSC-CM. The CM was centrifuged at 3,000 rpm/min for 10 minutes at 4°C to remove the cell debris and then was concentrated 20-fold by dialysis in a bag of a tangential flow microfiltration membrane (4-kDa molecular weight cutoff; Sartorius, GER) at 4°C.

### 2.4. ELISA

Wnt7a levels were measured by enzyme-linked immunosorbent assay (ELISA) according to the manufacturer's instructions (BioTek, VT, USA).

### 2.5. Preparation, Isolation, and Cultivation of Fibroblasts and Keratinocytes

Normal human skin keratinocytes and human fibroblasts (HFs) were derived from adult skin obtained from surgical waste, as previously described [25, 26]. Fibroblasts obtained from the outgrowth of explant cultures were grown in DMEM (Gibco, NY, USA) supplemented with 10% fetal bovine serum (Gibco, NY, USA), and cells from passage 3 were used for the experiments. Keratinocytes were cultured in the EpiLife medium (Gibco, NY, USA) at 37°C in a humidified atmosphere containing 5% of CO_2_. The medium was replaced every 2 to 3 days.

### 2.6. Preparation of the CM of Fibroblasts

The culture supernatant was collected from HFs (passage 3) as HF-CM. We used MSC-CM and Wnt-CM for the treatment of fibroblasts for 24 hours after removal of the supernatant; we then added a fresh culture medium after 48 hours and collected the supernatant to prepare HF^MSC-CM^-CM and 20-fold concentrated HF^Wnt-CM^-CM.

### 2.7. The Use of the CM In Vitro

After cultivation of fibroblasts or keratinocytes, the CM (20-fold concentrated) was added to DMEM (the CM and DMEM were mixed in the 1 : 10 ratio, v/v).

### 2.8. RNA Isolation and Real-Time RT-PCR

Total RNA was isolated using the RNeasy Mini Kit (Qiagen, CA, USA). Single-stranded cDNA was synthesized using SuperScript II reverse transcriptase and oligo (dT) (Invitrogen, CA, USA). Quantitative RT-PCR was used to measure transcription of genes, and the data were normalized to glyceraldehyde 3-phosphate dehydrogenase (GAPDH). PCR was run on an ABI 7500 Real-Time PCR System (Applied Biosystems, USA) under the following cycling conditions: one cycle of 95°C for 10 min and 40 cycles of 95°C for 15 s and 60°C for 1 min, with the primers shown in Table 1. Relative mRNA expression was calculated by 2^−ΔΔCt^ method.

### 2.9. Western Blot Analysis

Western blotting was performed as previously described [27]. Total protein was isolated from MSCs and from retrovirally infected MSCs. Primary antibodies were a rabbit polyclonal antibody to Wnt7a, c-Myc, K6, and K16 and a mouse monoclonal antibody to GAPDH (Abcam, MA, UK). The secondary antibodies were goat anti-rabbit and goat anti-mouse IgG antibodies (horseradish peroxidase-conjugated; Abcam, MA, UK). The blots were analyzed by densitometry in the ImageJ software (NIH, MD, USA).

### 2.10. Immunocytochemistry

For the immunocytochemical analysis, cells were fixed in 4% paraformaldehyde for 30 min at 4°C and then permeabilized by 0.1% Triton X-100 for 15 min, followed by blocking with 3% fetal bovine serum for 30 min. Primary antibodies: rabbit polyclonal antibodies to Wnt7a and *α*-SMA (Abcam, MA, UK) were incubated with the cells overnight at 4°C. Secondary antibodies: an Alexa Fluor 594-conjugated goat anti-rabbit IgG antibody and an Alexa Fluor 488-conjugated goat anti-mouse IgG antibody (Invitrogen, CA, USA) were added to the cells and incubated for 1 h at 37°C. Finally, the cells were stained with Hoechst (Vector, Burlingame, CA) at a 1 : 3000 dilution and were examined under a fluorescence microscope (Olympus BX53, JP).

### 2.11. The Animal Model

We used 6-week-old C57BL/6 mice as a model of the full-thickness skin injury. After anesthesia, 1 cm diameter wounds were created on the back skin of the mice; then, 100 *μ*L of Wnt-CM, MSC-CM, DMEM (Gibco BRL, NY, USA), HF^MSC-CM^-CM, or HF^Wnt-CM^-CM was injected at multiple points into the wound area.

### 2.12. Histological Analysis

The wounded skin from each mouse was collected for histological analysis. The samples were fixed in 10% formalin and then embedded in paraffin blocks to prepare paraffin sections (4 *µ*m thick). The general histological features were visualized by hematoxylin and eosin (H&E) staining and were examined under a microscope (Olympus BX53JP).

### 2.13. The Cell Scratch Experiment

Fibroblasts and keratinocytes were seeded in 6-well plates at 5 × 10^5^/well. When the cells covered the bottom of the well, a scratch was made with a sterilized P1000 pipette tip (width 1.0–1.2 mm), and the cells then were cultured in different media after a wash with PBS. After 24 h, the scratches were analyzed by microscopy (Olympus BX53, JP), and the scratch area was measured in the IPP software (Media Cybernetics).

### 2.14. Statistics

The results are expressed as mean ± SEM. All experiments were repeated three times with independent cultures, and similar results were obtained. Statistical significance was assessed using Student's* t-*test. Statistical significance was assumed for *P* values <0.05 or <0.01.

## 3. Results

### 3.1. Wnt7a Overexpression in UC-MSCs

**Figure 1:**
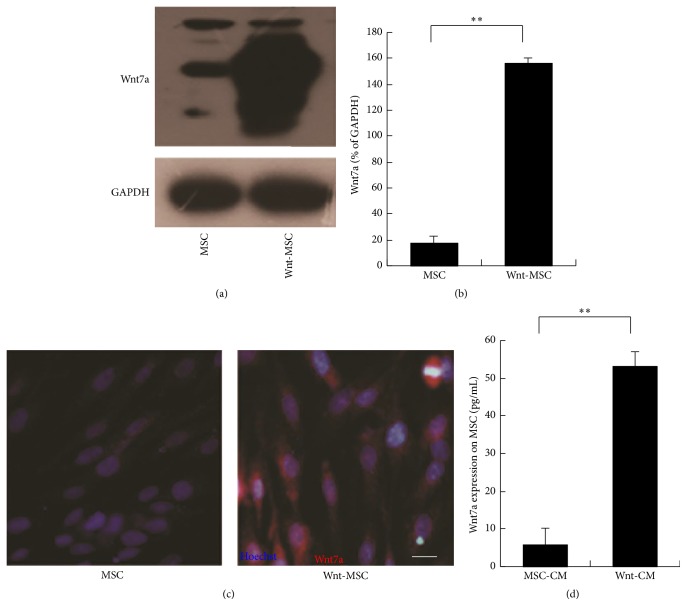
Wnt7a expression in human MSCs. (a, b) Western blots of Wnt7a and corresponding semiquantitative analysis. (c) Immunocytochemical analysis of MSCs overexpressing Wnt7a. The nuclei were counterstained with Hoechst. (d) Wnt7a was quantified in the CM by ELISA. The data are presented as mean ± SEM, *n* = 3; ^*∗∗*^*P* < 0.01. In the images, Wnt7a (red fluorescence) and Hoechst (blue fluorescence) staining are shown. The scale bar in panel (b) is 20 *μ*m.

A retroviral vector was constructed to express Wnt7a and was propagated in HEK293 cells. We purified the retrovirus to use it for the induction of Wnt7a expression in UC-MSCs by means of polybrene exposure. The retrovirus-mediated transduction of Wnt7a into UC-MSCs was confirmed by Western blotting and immunocytochemistry. Expression levels of the Wnt7a protein were higher in these UC-MSCs (Figures 1(a) and 1(b)). The immunocytochemical analysis also showed that the retrovirus-mediated transduction of Wnt7a into UC-MSCs made these cells positive for Wnt7a expression (Figure 1(c)). We detected the expression of Wnt7a in the culture supernatant by enzyme-linked immunosorbent assay (ELISA). The results showed that the level of secreted Wnt7a protein (as a result of the retroviral-mediated transduction of Wnt7a into UC-MSCs) was significantly increased as compared to that in the control group (Figure 1(d)). These data showed that Wnt7a was successfully expressed in UC-MSCs and was secreted into the supernatant of the cultured UC-MSCs.

### 3.2. Wnt-CM Enhances Healing of Full-Thickness Skin Wounds in Mice

**Figure 2:**
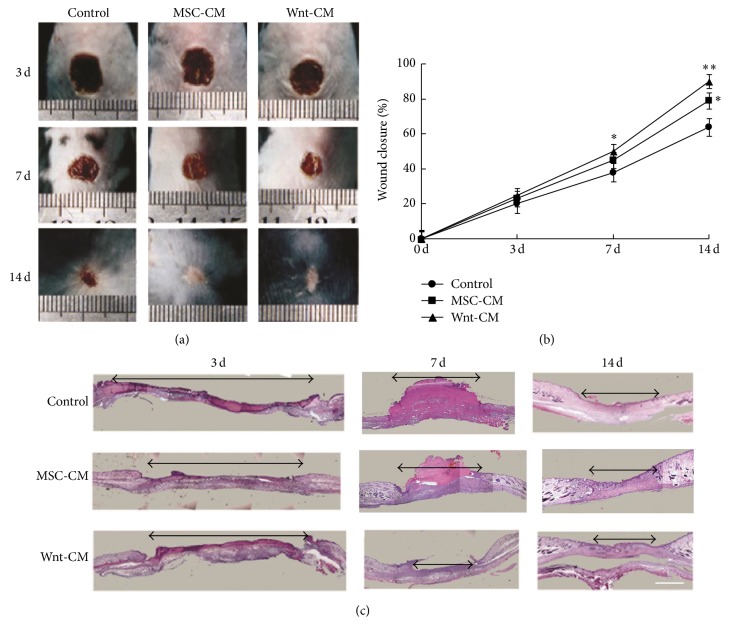
Topical injection of Wnt-CM accelerated healing of full-thickness skin wounds in mice. (a) Typical images of dorsal skin showing effectiveness of wound closure in the mice of treatments groups DMEM, MSC-CM, and Wnt-CM. (b) The percentage of wound closure on days 3, 7, and 14 after the surgical injury. (c) On postinjury days 3, 7, and 14, healing skin was examined by H&E staining of the tissue slices. The data are presented as mean ± SEM, *n* = 3; ^*∗*^*P* < 0.05 and ^*∗∗*^*P* < 0.01. The double arrow line denotes the wound area. The scale bar is 500 *μ*m in panel (c).

A model of healing of a full-thickness skin wound was used to estimate the effects of topical application of Wnt-CM on wound healing. We collected the culture medium from Wnt7a-overexpressing UC-MSCs and prepared Wnt-CM through tangential flow concentration (20-fold). Then, 100 *μ*L of Wnt-CM was injected subcutaneously at multiple points into the wound area of each mouse, with the same dose of MSC-CM or Dulbecco's modified Eagle's medium (DMEM) serving as control. The wound closure rates in the Wnt-CM-treated mice on postinjury days 3, 7, and 14 were 25.3%, 51.6%, and 91.5%, respectively (*P* < 0.05, *n* = 3), whereas in MSC-CM-treated mice they were 22.9%, 44.3%, and 76.3% (*P* < 0.05, *n* = 3), and in DMEM-treated mice these rates were 20.1%, 38.7%, and 65.1% (*P* < 0.05, *n* = 3; Figures 2(a) and 2(b)). The results showed that Wnt-CM significantly enhanced the closure rates in comparison with MSC-CM, but the MSC-CM treatment group showed better results than did the DMEM treatment group. Similarly, H&E-stained histological slices of skin samples showed similar closure rates on postinjury days 3, 7, and 14 (Figure 2(c)). Thus, the results showed that Wnt-CM can enhance wound repair in mice.

### 3.3. Wnt-CM Enhances Expression of ECM Components and Migration of Fibroblasts

**Figure 3:**
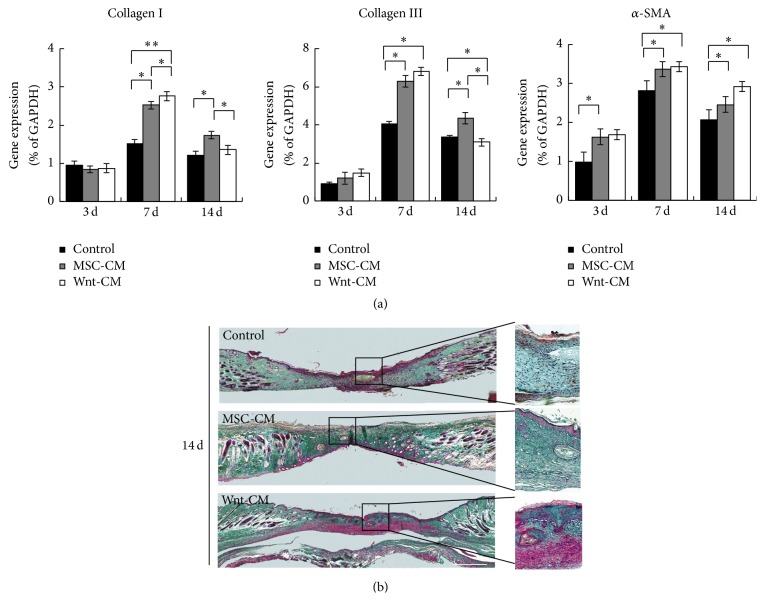
Expression of ECM components during the wound healing process in mice. (a) Expression levels of *α*-SMA, collagen III, and collagen I mRNA in mice of treatment groups DMEM, MSC-CM, and Wnt-CM were analyzed by real-time PCR and normalized to that of GAPDH. (b) Representative images of skin biopsy samples (obtained from mice of treatment groups DMEM, MSC-CM, and Wnt-CM), showing staining with Masson's trichrome. Statistical analysis involved unpaired* t* tests. The data are presented as mean ± SEM, *n* = 3; ^*∗*^*P* < 0.05 and ^*∗∗*^*P* < 0.01. The scale bar is 200 *μ*m in panel (b).

Injury to the skin resulting in a wound is known to trigger a repair process that is characterized by major alterations in both the composition and structure of the ECM. Accordingly, mRNA expression levels of ECM-related molecules were then evaluated. Compared to the DMEM treatment group, the MSC-CM and Wnt-CM treatment groups expressed significantly larger amounts of ECM molecules in the wound area, including *α*-SMA, collagen I, and collagen III (Figure 3(a)). To evaluate collagen accumulation in the skin, we used Masson's trichrome staining. The results showed that Wnt-CM markedly increased collagen deposition in the dermis compared with MSC-CM and DMEM (Figure 3(b)). Therefore, these findings indicate that Wnt-CM efficiently enhances wound healing-related events, such as collagen production.

**Figure 4:**
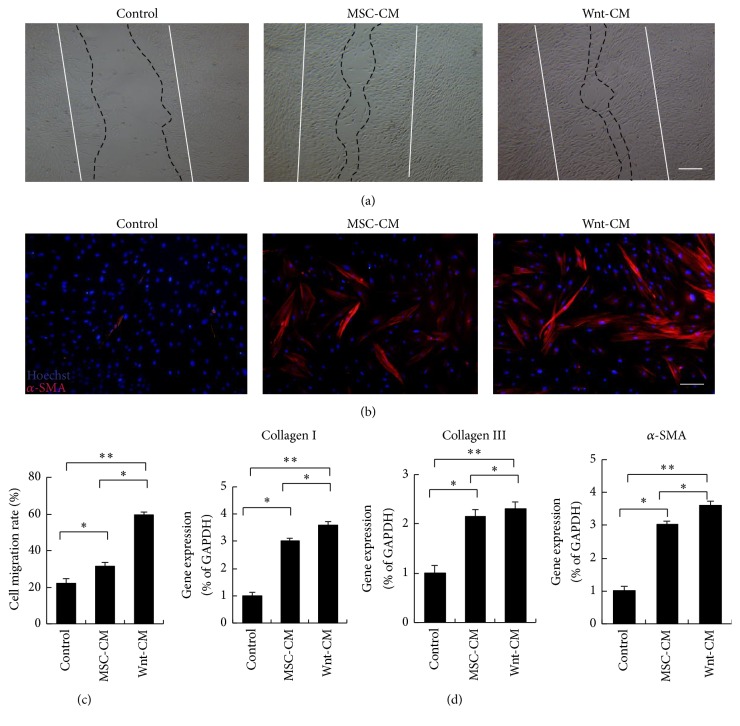
Wnt-CM stimulates migration and promotes expression of *α*-SMA and ECM components in fibroblasts. (a) Wnt-CM enhanced migration of primary human fibroblasts in a wound scratch assay (after 24 h) as compared to MSC-CM and DMEM. (b) Immunocytochemical staining for *α*-SMA shows upregulation of *α*-SMA induced by Wnt-CM. (c) Quantification of optical density of the immunocytochemical staining for *α*-SMA. (d) Expression levels of *α*-SMA, collagen III, and collagen I mRNA after treatment with DMEM, MSC-CM, or Wnt-CM were analyzed by RT-PCR and normalized to that of GAPDH. The data represent mean ± SEM, *n* = 3; ^*∗*^*P* < 0.05 and ^*∗∗*^*P* < 0.01. Solid lines indicate the initial wound area; dotted lines demarcate the migrating front of the cells. The scale bar is 100 *μ*m in panel (a) and 50 *μ*m in panel (b).

The functional role of Wnt-CM in fibroblasts was analyzed in vitro. Human dermal fibroblasts (HFs) were obtained from surgical waste (adult skin samples) and were incubated with DMEM, MSC-CM, or Wnt-CM. A scratch wound assay showed that Wnt-CM accelerated migration of HFs into the wound area in comparison with MSC-CM and DMEM at 24 hours after scratching (Figures 4(a) and 4(c)). Therefore, we next tested the expression of migration-related genes of fibroblasts. We found that Wnt-CM can significantly increase expression of the smooth muscle cell actin isoform called -smooth muscle actin (*α*-SMA), collagen I, and collagen III in HFs as compared to DMEM and MSC-CM (Figures 4(b) and 4(d)). Overall, these results indicate that Wnt-CM significantly enhanced migration and ECM expression of fibroblasts.

### 3.4. Wnt-CM Enhances Reepidermalization during Skin Healing in Mice

**Figure 5:**
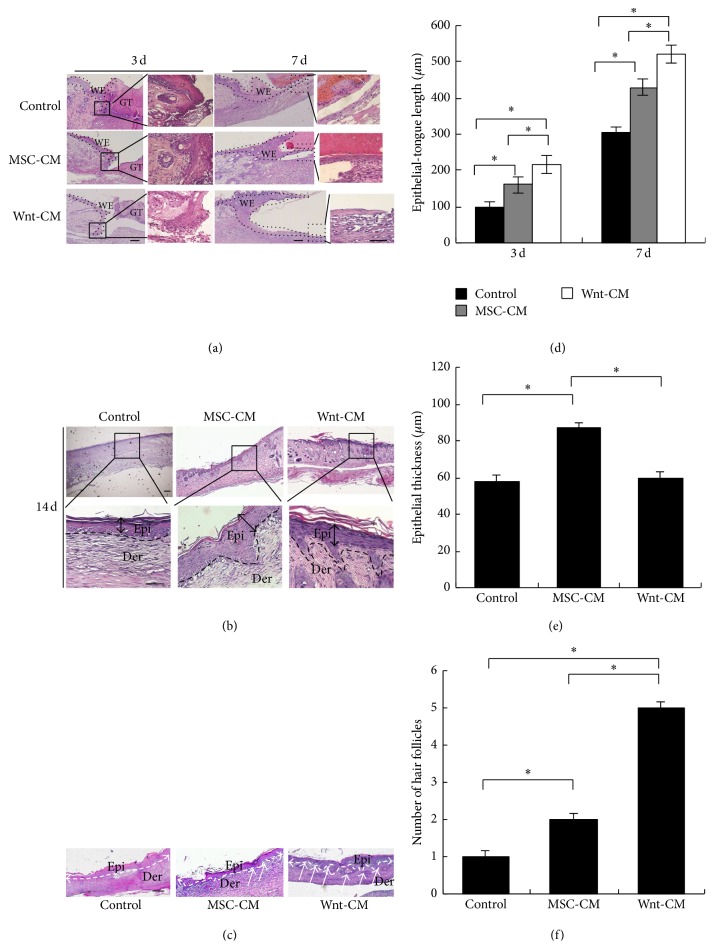
Histomorphological analysis of reepidermalization during skin healing in mice. (a, d) Histological analysis of wounds showed significantly increased length of the wound epithelial tongue in mice of treatment group Wnt-CM, as compared to the MSC-CM and DMEM treatment groups on postinjury days 3 and 5. (b, e) The epidermis thickness of the wounds treated with Wnt-CM, MSC-CM, or DMEM in mice on postinjury day 14. (c) The number of regeneration hair follicles in a wound. The data are presented as mean ± SEM, *n* = 3; ^*∗∗*^*P* < 0.01. The arrow indicates hair follicle regeneration. GT, granulation tissue; WE, wound epithelium; Epi, epidermis; Der, dermis. The scale bar in panels (a), (b), and (c) is 100 *μ*m.

To elucidate the effects of Wnt-CM on reepidermalization during skin healing, epithelial-tongue length and epidermal thickness of skin wounds were examined by histomorphological analysis on postinjury days 3, 7, and 14. The Wnt-CM treatment group yielded significantly better results than did the MSC-CM and DMEM groups on postinjury days 3 and 7, but the MSC-CM group showed better results than did the DMEM group (Figures 5(a) and 5(d)). Mice treated with Wnt-CM had a thicker epidermis with more organized cell layers as compared to the DMEM group on postinjury day 14 (Figures 5(b) and 5(e)). The Wnt-CM treatment group also showed regeneration of more hair follicles as compared to the MSC-CM treatment group (Figures 5(c) and 5(f)). These results confirmed that Wnt-CM can substantially increase epidermal thickness and the number of migrating cells in the epithelial-tongue area.

### 3.5. The Inhibitory Effects of Wnt-CM on Keratinocyte Migration Are Mediated by c-Myc

**Figure 6:**
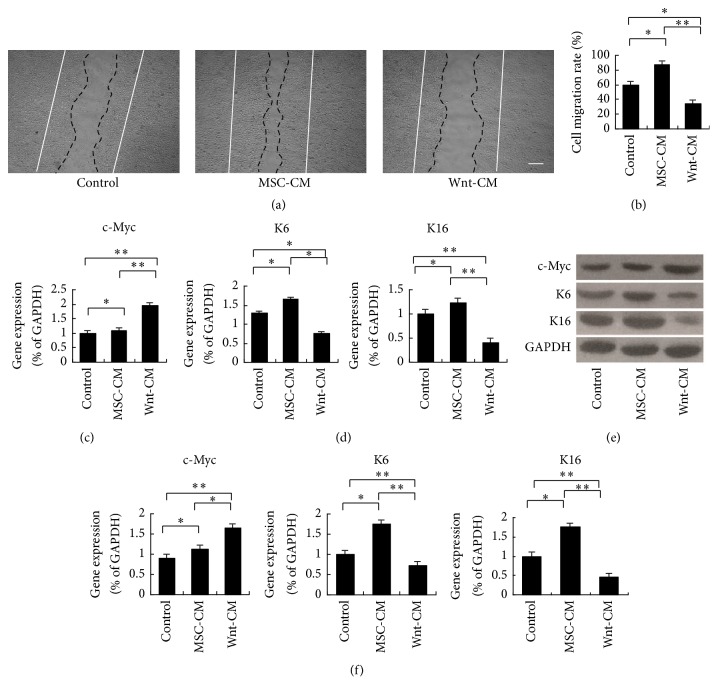
Wnt-CM inhibits migration and expression of the cytoskeletal components K6 and K16 via upregulation of c-Myc in human keratinocytes. (a and b) Wnt-CM inhibited migration of human keratinocytes in the wound scratch assay after 24 h as compared to MSC-CM and DMEM. (c) Expression levels of c-Myc mRNA after treatment with Wnt-CM, MSC-CM, or DMEM were analyzed by real-time PCR and normalized to that of GAPDH. (d) Expression levels of K6 and K16 mRNA after treatment with Wnt-CM, MSC-CM, and DMEM were analyzed by RT-PCR and normalized to that of GAPDH. (e, f) Western blots analysis of c-Myc, K6, K16, and corresponding semiquantitative analysis. The data represent mean ± SEM, *n* = 3; ^*∗*^*P* < 0.05 and ^*∗∗*^*P* < 0.01. Solid lines indicate the initial wound area; dotted lines demarcate the migrating front of the cells. The scale bar in panel (a) is 100 *μ*m.

To demonstrate the effects of Wnt-CM on keratinocyte migration, scratch wound assays were performed. Keratinocyte migration was significantly retarded by Wnt-CM treatment as compared to either MSC-CM or DMEM treatment at 24 hours after scratching (Figures 6(a) and 6(b)). c-Myc can impair migration of basal keratinocytes and inhibit wound healing [23]. Accordingly, we assessed mRNA and protein expression levels of c-Myc at 48 hours (Figures 6(c) and 6(e)). Our results showed that c-Myc expression in the wound area was significantly higher in the Wnt-CM treatment group than in the MSC-CM and DMEM treatment groups at 48 hours. Next, the cytoskeletal components important for migration (K6 and K16) were analyzed by RT-PCR and Western blot. Wnt-CM significantly inhibited the expression of K6 and K16 at 48 h after scratching (Figures 6(d)–6(f)). These results suggested that Wnt-CM inhibits the migratory ability and cytoskeletal organization of keratinocytes by increasing the expression of c-Myc.

### 3.6. Wnt-CM Causes Fibroblasts to Promote Keratinocyte Migration and Expression of Cytoskeletal Proteins

**Figure 7:**
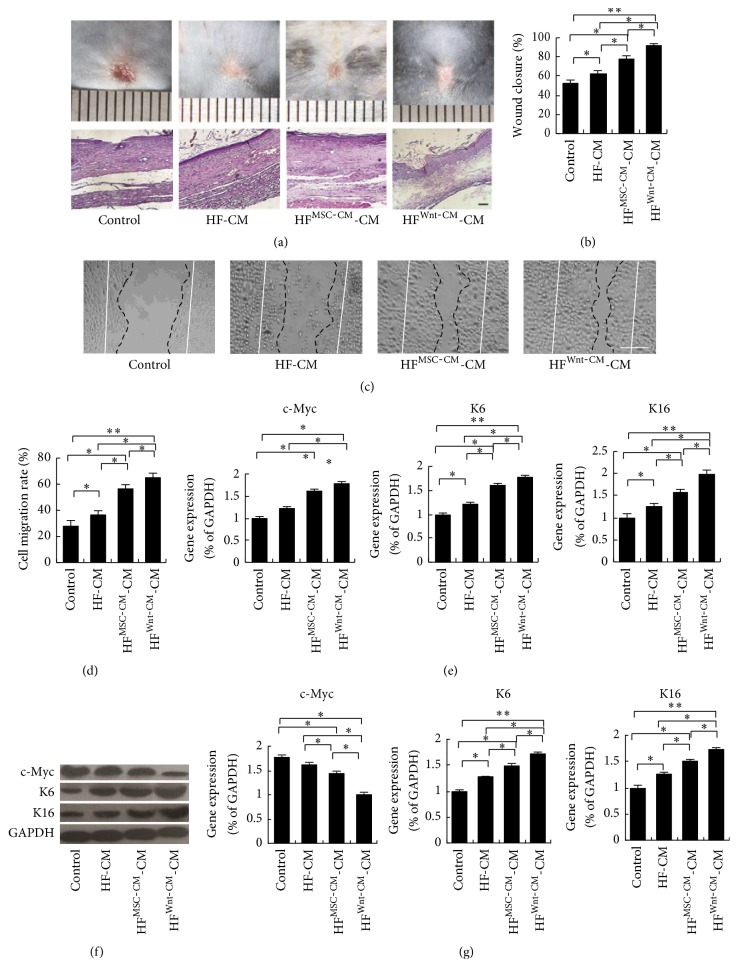
HF^Wnt-CM^-CM enhances migration and expression of the cytoskeletal components K6 and K16 in human keratinocytes. (a) Typical images of dorsal skin showing effectiveness of wound closure in the mice and H&E-stained histological slices of treatments groups HF^Wnt-CM^-CM, HF^MSC-CM^-CM, and HF-CM. (b) Evaluation of wound closure by morphometrical analysis of wound areas (ImageJ, NIH). (c and d) HF^Wnt-CM^-CM enhanced migration of human keratinocytes in a wound scratch assay after 24 h as compared to HF^MSC-CM^-CM, HF-CM, and DMEM and corresponding semiquantitative analysis data. (e) Expression levels of c-Myc, K6, and K16 mRNA after treatment with HF^Wnt-CM^-CM, HF^MSC-CM^-CM, or HF-CM were analyzed by real-time PCR and normalized to that of GAPDH. (f, g) Western blots analysis of c-Myc, K6, K16, and corresponding semiquantitative analysis. The data represent mean ± SEM, *n* = 3; ^*∗*^*P* < 0.05 and ^*∗∗*^*P* < 0.01. Solid lines indicate the initial wound area; dotted lines demarcate the migrating front of the cells. The scale bar is 100 *μ*m.

The early stage of wound healing is infiltration of fibroblasts into the damaged area where they synthesize collagen and form the ECM; the crosstalk between fibroblasts and keratinocytes facilitates wound closure. Therefore, we tested whether treatment of fibroblasts with Wnt-CM can affect the skin wound and keratinocyte migration. We used HF^MSC-CM^-CM or HF^Wnt-CM^-CM (treatment of fibroblasts with MSC-CM or Wnt-CM for 24 hours after removal of the supernatant; a fresh culture medium was added and the supernatant was collected after 48 hours) to treat the wound and cultured keratinocytes; the CM from nonactivated fibroblasts (HF-CM) and DMEM served as controls. We found that HF^Wnt-CM^-CM significantly enhanced the closure rates (Figures 7(a) and 7(b)) and promoted keratinocyte migration and cytoskeletal-protein expression in comparison with HF^MSC-CM^-CM, HF-CM, and DMEM (Figures 7(c)–7(g)). In contrast, HF^Wnt-CM^-CM did not influence the expression of c-Myc according to RT-PCR and Western blot analysis of cultured keratinocytes (Figures 7(e)–7(g)). On the other hand, we found that HF^Wnt-CM^-CM could not promote regeneration of hair follicles by H&E-stained histological slices analysis (Figure 7(a)). Thus, these results showed that Wnt-CM accelerates wound repair and enhances migration of keratinocytes by activating fibroblasts.

## 4. Discussion

In this study, we explored the effects of Wnt signaling in combination with UC-MSCs on wound repair. We found that Wnt-CM can promote wound repair and induce hair follicle regeneration. Moreover, Wnt-CM by activating fibroblasts enhanced secretory expression of ECM components, which interacted with keratinocytes to promote keratinocyte migration and to enhance reepidermalization. Moreover, our data indicate that Wnt-CM can enhance the crosstalk between cells and among cells in the complex wound microenvironment, thereby enhancing these multifaceted wound healing activities.

MSCs are increasingly studied as a potential treatment method for clinical application [28–30]. Recent studies showed that treatment of cutaneous wounds with MSCs accelerates wound healing kinetics, increases epithelialization, and promotes angiogenesis. Although MSCs may repair damaged tissue by direct induction of differentiation, they also have a second function: secretion of a broad repertoire of trophic factors and immunomodulatory cytokines [31, 32]. A large body of work has shown that MSCs can improve wound healing through adjustment of the local microenvironment by secreting various factors [33, 34]. Moreover, Wnt proteins are key mediators of wound regeneration and participate in various related processes, from the development of the dermis to formation of skin appendages [20]. Wnts can drive epidermal-mesenchymal interactions that enhance wound-induced follicle neogenesis in mouse models, and they are also important for maintenance of the hair induction activity of cultured DP cells [13, 35–37]. Overexpression of Wnt signaling molecules promotes cellular proliferation, migration, and ECM degradation; these changes reflect the basic phases of wound regeneration [38]. In this study, we injected Wnt-CM into skin wounds of mice and observed markedly accelerated wound closure and hair regeneration compared to those in mice that received injection of MSC-CM and a control group. MSC-CM can also accelerate wound closure and increase expression of ECM components in comparison with the DMEM treatment group, but MSC-CM causes significantly weaker regeneration of hair follicles as compared to Wnt-CM. The results proved that the synergy of MSCs with a Wnt protein can enhance wound healing and hair follicle regeneration.

Regeneration of hair follicles during wound repair is similar to the process of hair follicle initiation in the embryonic period. Various researchers have demonstrated that hair follicle neogenesis can be induced in a large wound through the overexpression of Wnt7a in the microenvironment [13]. We previously reported that Wnt-CM can induce regeneration and accelerate the cycling of hair follicles by activating DP cells in mice [39]. At the same time, Wnt-CM can maintain the ability of DP cells to induce hair regeneration when cotransplanted with epidermis cells in nude mice [37]. Here, we prepared CM from the culture supernatant of Wnt7a-overexpressing UC-MSCs (Wnt-CM) and used this CM to treat skin wounds in a mouse model. Newly formed hair follicles were observed in the basal epithelial tissues as well as an elevated number of hair follicles after Wnt-CM treatment as compared to the control group. Therefore, we can conclude that Wnt-CM stimulates wound healing and promotes hair follicle regeneration by stimulating the Wnt signing pathway.

Healing of full-thickness wounds involves migration of keratinocytes, fibroblasts, and endothelial cells to the wound bed prepared with deposition of appropriate ECM molecules and proliferation of these cells [5, 40]. Fibroblasts are the major stromal cells in the dermis and in many other tissues. They appear in the wound healing process and release numerous cytokines inducing production of ECM components and contributing to wound closure [41]. Our data here showed that Wnt-CM significantly promotes wound repair and expression of ECM molecules. In addition, Wnt-CM and MSC-CM (separately) can induce expression of *α*-SMA by fibroblasts in vitro. Nonetheless, the *α*-SMA expression implies that fibroblasts acquire the myofibroblastic phenotype [42]. Myofibroblasts perform a pivotal function in organogenesis during mesenchymal-epithelial interactions and accelerate wound healing by secreting components of the ECM and of the basement membrane [43]. Thus, our results suggest that Wnt-CM and MSC-CM can enhance ECM secretion and accelerate wound contraction.

Keratinocytes are a major cellular component of the epidermis during a normal wound healing process [10]. Research has shown that the overexpression of *β*-catenin and c-Myc impairs wound healing by inhibiting migration of keratinocytes and by altering their differentiation [23, 44]. Our results also confirmed that Wnt-CM inhibits cytoskeletal organization of keratinocytes and their migratory ability. In contrast, the results of the in vivo experiment showed that Wnt-CM increases reepidermalization during skin healing in mice. Thus, we can conclude that Wnt-CM may cause fibroblasts to promote reepithelialization. We found that HF^Wnt-CM^-CM can promote migration of keratinocytes and expression of cytoskeletal proteins. We observed that this CM can promote wound healing in mice, but the number of regenerated hair follicles was not increased significantly. Consequently, we believe that HF^Wnt-CM^-CM can effectively enhance wound healing and keratinocyte migration but cannot induce hair follicles without exogenous Wnt proteins. Therefore, Wnt proteins may be a useful and selective therapeutic target for hair regeneration during wound repair. Thus, coordinated expression of multiple genes in MSCs holds promise for stem cell-based therapy. Synergistic effects of treatment with Wnt7a and MSCs yield better results than MSC-CM treatment alone.

MSC therapy is a promising method to overcome the limitations of current therapies. Strategies to increase the efficacy of MSC properties through the use of gene engineering to enhance the natural production of specific proteins or to make their own requires the expression of protein, which can greatly broaden the spectrum of disease that can be treated and fully exploit their potential [45]. Enhancing the abilities of these cells to migrate, survive, and promote healing through immunomodulation, differentiation, and angiogenesis is the goal [46]. However, the mechanisms of action include two main aspects: (1) MSC only as a vehicle for transferring genes and drug delivery, but not to change the phenotypic and differentiation potential of MSC by using genetic modification [47] and (2) genes modified by MSC to enhance the expression of purpose genes and at the same time interact with cytokines, changing the secretion level to enhance the function [46, 48]. Thus, what role stem cells will play is something we need to continue to explore. Once these problems are solved, the coordinated expression of more genes in MSCs using complex expression systems shows promise for clinical therapy.

## 5. Conclusion

In summary, Wnt-CM can enhance secretion of ECM components and migration of fibroblasts and then promote wound healing and induce hair regeneration. HF^Wnt-CM^-CM also promotes wound healing and migration of keratinocytes, but, due to the lack of Wnt signals, the number of regenerated hair follicles is not increased. We believe that wound repair requires crosstalk among cells and between cells and cytokines, and any change in these processes will alter wound repair. Thus, the overexpression of Wnt7a by UC-MSCs can effectively accelerate wound healing and induce hair follicle regeneration by improving the wound microenvironment.

## Figures and Tables

**Table 1:** List of sequences of forward and reverse primers.

Genes	Species	Forward primer	Reverse primer
*α*-SMA	Human	5′-CTATGAGGGCTATGCCTTGCC-3′	5′-GCTCAGCAGTAGTAACGAAGGA-3′
Collagen I	Human	5′-GTGCGATGACGTGATCTGTGA-3′	5′-CGGTGGTTTCTTGGTCGG-3
Collagen III	Human	5′-TTGAAGGAGGATGTTCCCATCT-3′	5′-ACAGACACATATTTGGCATGGT-3′
K6	Human	5′-ACGGAAACTACTACGGCGAC-3′	5′-GGCCTTCGTATCCACAGCAC-3′
K16	Human	5′-GACCGGCGGAGATGTGAAC-3′	5′-CTGCTCGTACTGGTCACGC-3′
C-Myc	Human	5′-AATAGAGCTGCTTCGCCTAGA-3′	5′-GAGGTGGTTCATACTGAGCAAG-3′
GAPDH	Human	5′-CTGGGCTACACTGAGCACC-3′	5′-AAGTGGTCGTTGAGGGCAATG-3′
*α*-SMA	Mouse	5′-GTCCCAGACATCAGGGAGTAA-3′	5′-TCGGATACTTCAGCGTCAGGA-3′
Collagen I	Mouse	5′-GCTCCTCTTAGGGGCCACT-3′	5′-CCACGTCTCACCATTGGGG-3′
Collagen III	Mouse	5′-CTGTAACATGGAAACTGGGGAAA-3′	5′-CCATAGCTGAACTGAAAACCACC-3′
GAPDH	Mouse	5′-AGGTCGGTGTGAACGGATTTG-3′	5′-TGTAGACCATGTAGTTGAGGTCA-3′

PCR amplification conditions on the Applied Biosystems 7500 Real-Time PCR System: 58°C for 5 minutes; 95°C for 2 minutes; 40 cycles of 95°C for 15 seconds and 60°C for 60 seconds.
